# Inspiratory muscle training improves heart rate variability and respiratory muscle strength in obese young adults

**DOI:** 10.1371/journal.pone.0329623

**Published:** 2025-08-20

**Authors:** Piangkwan Sa-nguanmoo, Sainatee Pratanaphon, Arisa Parameyong, Jitapa Chawawisuttikool, Krekwit Shinlapawittayatorn, Nipon Chattipakorn, Siriporn C. Chattipakorn

**Affiliations:** 1 Department of Physical Therapy, Faculty of Associated Medical Sciences, Chiang Mai University, Chiang Mai, Thailand; 2 Integrated Neuro-Musculoskeletal, Chronic Disease, and Aging Research Engagement Center (I-CARE Center), Department of Physical Therapy, Faculty of Associated Medical Sciences, Chiang Mai University, Chiang Mai, Thailand; 3 Neurophysiology Unit, Cardiac Electrophysiology Research and Training Center, Faculty of Medicine, Chiang Mai University, Chiang Mai, Thailand; 4 Cardiac Electrophysiology Unit, Department of Physiology, Faculty of Medicine, Chiang Mai University, Chiang Mai, Thailand; 5 Center of Excellence in Cardiac Electrophysiology Research, Chiang Mai University, Chiang Mai, Thailand; 6 Department of Oral Biology and Diagnostic Sciences, Faculty of Dentistry, Chiang Mai University, Chiang Mai, Thailand; Japanese Academy of Health and Practice, JAPAN

## Abstract

This study determined the effect of inspiratory muscle training (IMT), a non-pharmacological treatment on pulmonary function, inspiratory muscle strength and autonomic modulation measured by heart rate variability in obese young adults. The study hypothesized that IMT improves inspiratory muscle strength and enhances autonomic modulation in obese young adults. Nineteen obese young adults (body mass index (BMI) ≥ 25 kg/m², according to the Asian-Pacific cutoff points), aged 18–25 years, were assigned to either a control group (n = 9) or an inspiratory muscle training (IMT) group (n = 10). The IMT group underwent a training load of 55% of maximum inspiratory pressure (MIP) and the control group had no load. Both groups performed 30 breaths twice a day, 5 days/week for 4 weeks. IMT load was readjusted weekly. Pulmonary function, inspiratory muscle strength and heart rate variability (HRV) were measured at baseline, and weeks 2 and 4. By week 2, inspiratory muscle strength was significantly improved in the IMT group (*p *< 0.05). The low-frequency/high-frequency (LF/HF) ratio was also reduced, suggesting improved sympathovagal balance. These changes indicate potential cardiovascular benefits of IMT in this population. However, no differences in pulmonary function or inspiratory muscle endurance were found between the groups. These findings suggested that IMT improves heart rate variability and increases inspiratory muscle strength in obese young adults.

## Introduction

Obesity has become a serious public health problem in all age groups, and the incidence is now rising in young adults [1]. The condition itself exerts numerous detrimental effects on the respiratory system, leading to issues such as dyspnea, obstructive sleep apnea syndrome (OSAS) [2], chronic obstructive pulmonary disease (COPD) [3] and asthma [4]. Impaired respiratory function significantly elevates mortality and morbidity rates in individuals with obesity [5]. Previous studies have reported that increased BMI is associated with diminished pulmonary function, as demonstrated by a reduction in forced vital capacity (FVC) and forced expiratory volume in one second (FEV1) [6,7]. Furthermore, obesity adversely affects respiratory muscle performance by reducing respiratory muscle endurance and inspiratory muscle strength, as indicated by a decrease in maximal voluntary ventilation (MVV) and maximum inspiratory pressure (MIP), respectively [8,9]. Obesity can affect respiratory functions via several mechanisms: i) mechanical changes, via the excessive accumulation of adipose tissue deposited on and around respiratory organs, and ii) systemic inflammation as shown by an increase in numerous inflammatory cytokines, which are produced by adipocytes [10]. Fat deposition in the chest wall and abdominal cavity leads to a reduction in the movement of the diaphragm and chest wall compliance, resulting in limited lung ventilation, increased breathing workload, decreased respiratory muscle strength and increased respiratory muscle fatigue [11]. Moreover, obesity has been related to sympathovagal imbalance or dysregulation of autonomic function, as characterized by decreased parasympathetic and increased sympathetic activities [12]. A decline in lung function and sympathovagal imbalance have been linked to increased cardiovascular risk [13,14]. Therefore, alternative therapeutic strategies that can improve respiratory function and sympathovagal balance in the obese condition may provide beneficial effects regarding respiratory performance, cardiovascular function and quality of life in young adults with obesity.

It is well established that IMT is a practical, minimally invasive technique that can be performed at home [15], enhances not only respiratory muscle strength, but also respiratory muscle endurance in various populations [16–18]. Previous studies showed that long-term IMT for 12 weeks improved MIP and MVV in morbidly obese individuals [17] and improved autonomic modulation in patients with COPD [19]. Interestingly, a recent study showed that a 4-week period of IMT intervention significantly improved both inspiratory muscle strength and functional fitness by increasing the 6 minute walk distance in obese and overweight adults [20]. Although IMT has shown benefits across various populations, its effectiveness may differ among subgroups of obese young adults, depending on factors such as baseline autonomic function, degree of obesity, and presence of comorbid conditions. Additionally, a 4-week IMT intervention conferred beneficial results on cardiac autonomic modulation by enhancing sympathovagal balance in elderly women [21,22]. Although previous studies have demonstrated the beneficial effects of IMT on autonomic function, these findings were primarily reported in patients with heart failure or other chronic conditions [23,24]. Furthermore, while IMT improves functional capacity and autonomic regulation, it is not associated with significant weight reduction. For instance, a previous study reported no significant changes in body weight following IMT in heart failure patients [23]. Nevertheless, unmeasured weight fluctuations during the intervention period may act as potential confounders, particularly in HRV-related outcomes. To date, there is limited research on the autonomic effects of short-term IMT in obese young adults, despite their elevated cardiometabolic risk and the well-established association between obesity and reduced heart rate variability (HRV). Our study aims to address this gap by investigating whether a short-duration IMT program can improve sympathovagal balance in this specific population. Therefore, the current study aimed to test the hypothesis that a 4-week IMT program increases respiratory muscle strength and improves sympathovagal balance in young obese individuals.

## Materials and methods

### Study design

This study was an experimental design with age- and gender-matched pairs. The methodology was reviewed and approved by the Human Research Ethics Committee at Chiang Mai University’s Faculty of Associated Medical Sciences (Approval No. AMSEC-62EX-052) in accordance with the Declaration of Helsinki. Prior to participation, all individuals provided written informed consent. All procedures adhered to applicable standards and regulations. Participants were recruited from 15 October 2019–15 August 2020 via a public advertisement.

### Sample size determination

The sample size analysis was calculated using G*Power software, using data from our pilot study involving 10 obese participants. In the control and IMT groups of that pilot trial, the mean MIP was 100 and 130 cmH_2_O, respectively (pooled standard deviation: 30 cmH_2_O). Using a power of 0.8, a two-sided alpha level of 0.05, and an effect size 0.3, a minimum sample size of 20 participants was required.

### Study participants

**Fig 1:**
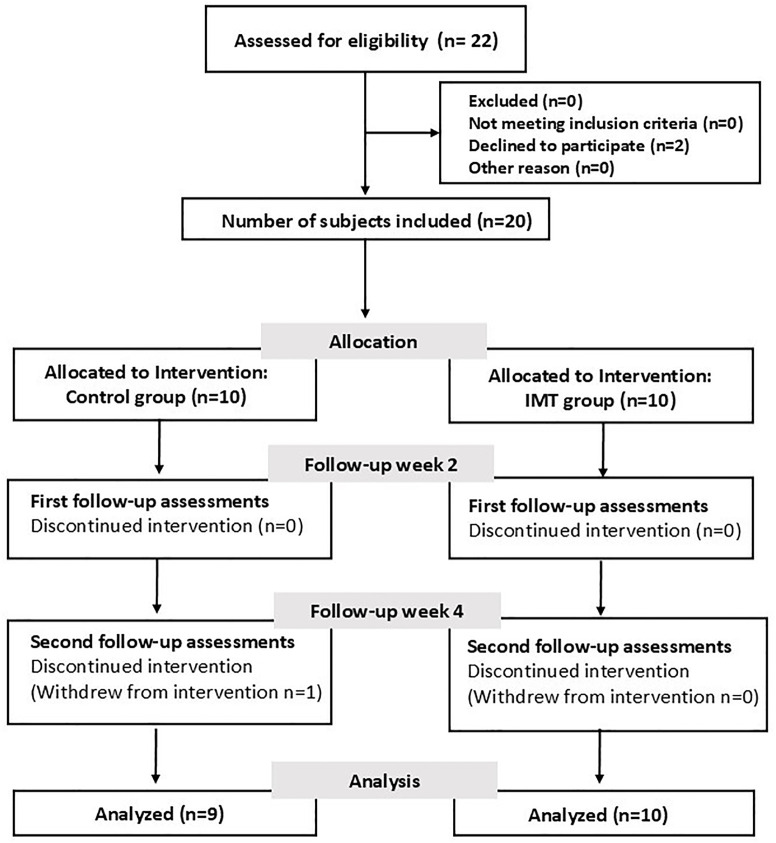
The experimental protocol of the study. IMT, inspiratory muscle training.

Twenty-two obese participants (BMI ≥ 25 kg/m², classified according to the Asian-Pacific cutoff points) [25], aged between 18 and 25 years, were included in this study. Participants who smoked, had underlying cardiopulmonary, neurological, or musculoskeletal diseases, or were unable to follow the protocol were excluded (n = 2). All participants completed baseline assessments of body composition, body mass, height, waist and hip circumference, pulmonary function, inspiratory muscle strength and cardiovascular autonomic function. After baseline data collection, eligible participants were randomly assigned to either the control group (Con, n = 10) or the inspiratory muscle training group (IMT, n = 10), matched by sex, age, and initial BMI. Assessments were repeated after 2 and 4 weeks of intervention. However, one male in the control group contracted COVID-19 during the intervention and withdrew due to illness, leaving 19 obese young adults who completed the study (Fig 1).

### Inspiratory muscle training

The IMT group followed a 4-week inspiratory muscle training (IMT) protocol using the POWERbreathe device (Gaiam, UK) at 55% of MIP, with the load adjusted weekly to maintain this percentage. The POWERbreathe device has been demonstrated to provide reliable and valid measurements in previous clinical trials involving inspiratory muscle training. All assessments were conducted by trained assessors following standardized protocols to minimize operator variability. A training intensity of 55% MIP was selected based on prior research demonstrating that this level induces improvements in inspiratory strength and functional performance over short durations [20]. The control group did not undergo any load. Both groups performed the program independently at home, completing 30 inspiratory exercises (5 sets of 6 breaths with 2 minutes rest between sets), twice daily, 5 days per week for 4 weeks. This protocol was chosen based on previous studies demonstrating improvements in inspiratory muscle strength and functional fitness [20]. Participants in the IMT group were instructed to maintain a daily training log, recording the number of sessions completed each day. In addition, weekly phone check-ins were conducted by research staff to encourage adherence and verify compliance.

### Anthropometrics

Body mass and composition were measured using an electrical impedance device (Tanita BC-418, Tokyo, Japan), while height was measured with a stadiometer. BMI was calculated by dividing body mass (kg) by height squared (m²), with a cutoff of 25 kg/m² for Asian populations [25]. Waist circumference was measured at the midpoint between the last rib and the iliac crest, and hip circumference was measured around the widest part of the buttocks [26].

### Pulmonary function

Pulmonary function tests, including FVC, FEV1, and FEV1/FVC ratio (expressed as percentages), were measured using a CHESTGRAPH HI-105 spirometer (Chest MI, Tokyo, Japan). The spirometer was calibrated daily using a 3-liter syringe according to ATS/ERS recommendations to ensure measurement accuracy [27], and the highest of three trials was used for data analysis.

### Maximal voluntary ventilation

MVV, indicating respiratory muscle endurance [28], was measured using a CHESTGRAPH HI-105 spirometer (Chest MI, Tokyo, Japan) following ATS/ERS protocols [29]. Participants inhaled and exhaled as quickly and deeply as possible for 12–15 seconds while seated. MVV values were expressed in L/min and as a percentage of predicted reference values.

### Inspiratory muscle strength

MIP, representing inspiratory muscle strength, was measured using a MicroRPM® device (MICRO Medical®, UK) following ATS/ERS protocols [29]. They performed maximal inspiratory efforts through a mouthpiece connected to the device, holding each effort for 1 second. The test was repeated three times, with the highest reading recorded for MIP. Measurements were taken while participants were seated.

### Heart rate variability analysis

Heart rate variability (HRV) is a clinical tool used to evaluate the balance between sympathetic and parasympathetic (vagal) modulation. All HRV recordings were obtained using the SEER Light Holter system, which is widely used in cardiovascular research and paired with validated MARS software version 7 for ECG analysis [30,31]. Fast-Fourier transform analysis was employed to assess both time and frequency domains. Time-domain measures included average heart rate, R-R intervals (NN), SDNN, SDANN, ASDNN, pNN50, and rMSSD. Frequency-domain analysis examined total power (0–0.4 Hz), high-frequency power (HF), low-frequency power (LF), and very-low-frequency power (VLF). All power spectral densities were reported in absolute units (ms²) to quantify autonomic nervous system activity.

### Statistical analysis

Data were expressed as Mean ± SD and analyzed using SPSS version 22.0 (IBM SPSS Statistics, IBM Corp., USA). The Shapiro–Wilk test assessed normality. Independent t-tests (and a chi-squared test for gender) were used to compare general characteristics between groups. Pulmonary function, MVV, MIP, and HRV changes from baseline to each time point were analyzed using 2 × 3 mixed model repeated measures ANOVA. Time (baseline, week 2, week 4) was the within-subject factor, and group (Control, IMT) was the between-subject factor. Post-hoc Bonferroni correction was applied for pairwise comparisons when interactions were significant. Statistical significance was set at p < 0.05.

## Results

A total of 22 obese young adults were recruited for this study, but two individuals declined to participate. Additionally, one was withdrawn from the intervention as he had a cold during the second follow up. Therefore, 19 obese young adults participated in this study. Although one participant withdrew due to illness, the remaining 19 participants still provided sufficient statistical power (estimated > 0.78) for detecting between-group differences in MIP, based on our pilot study and GPower analysis. Based on the log data, adherence to the IMT protocol was over 90% for all participants. There were no significant differences in age, height, body mass, BMI, waist circumference, hip circumference, waist-to-hip ratio, percentage of total body fat and truncal fat between the control and IMT groups, as summarized in Table 1.

**Table 1:** General characteristics of study population.

Parameters	Control (n = 9)	IMT (n = 10)	*p*-value
Age (years)	20.33 ± 2.00	20.00 ± 1.05	0.295
Gender (male/female)	5/4	6/4	0.998
Height (m)	1.69 ± 8.91	1.72 ± 8.72	0.267
Body mass (kg)	90.35 ± 10.73	95.23 ± 10.65	0.652
BMI (kg/m^2^)	31.62 ± 4.44	32.14 ± 5.54	0.679
Waist circumference (cm)	114.39 ± 15.05	116.95 ± 8.37	0.155
Hip circumference (cm)	105.67 ± 7.51	102.95 ± 4.06	0.600
Waist-to-hip ratio	0.91 ± 0.08	0.87 ± 0.08	0.917
Total body fat (%)	36.50 ± 11.62	35.40 ± 10.28	0.834
Truncal fat (%)	38.04 ± 11.87	36.62 ± 10.73	0.858

Data are represented as mean ± SD.

### Effects of inspiratory muscle training on lung function, respiratory muscle endurance and inspiratory muscle strength in obese young adults

All participants underwent lung function and inspiratory muscle strength testing. Lung function was assessed using spirometry. The results demonstrated that there were no significant differences in FVC, FEV1, FEV1/FVC ratio, %FVC, %FEV1, MVV, and %MVV between periods (baseline vs. weeks 2 vs. week 4) and groups (control vs. IMT), as shown in Table 2.

**Table 2:** Lung function prior to and following the 2 and 4-week intervention periods.

Parameters	Control group			IMT group			Mixed ANOVA	
	Baseline	Week 2	Week 4	Baseline	Week 2	Week 4	*p.* Inter	ES (partial η2)
FVC (L)	3.54 ± 0.97	3.48 ± 0.86	3.49 ± 0.95	4.03 ± 0.95	4.06 ± 1.06	4.08 ± 1.01	0.595	0.063
FVC %	94.05 ± 13.78	92.98 ± 21.96	89.37 ± 12.43	96.80 ± 11.51	102.35 ± 12.81	99.16 ± 17.41	0.457	0.093
FEV1 (L)	2.98 ± 0.85	2.94 ± 0.78	3.01 ± 0.80	3.53 ± 0.81	3.57 ± 0.91	3.66 ± 0.80	0.650	0.052
FEV1%	78.24 ± 15.46	79.22 ± 21.27	78.41 ± 14.65	87.34 ± 9.83	88.42 ± 12.26	87.67 ± 13.15	0.998	0.001
FEV1/FVC (%)	84.45 ± 10.06	85.65 ± 9.45	88.26 ± 10.63	88.02 ± 4.85	88.39 ± 4.62	88.32 ± 4.67	0.423	0.102
MVV (L)	91.76 ± 35.93	91.16 ± 23.62	88.73 ± 20.94	89.80 ± 31.45	95.21 ± 31.13	97.69 ± 20.68	0.455	0.094
MVV %	65.72 ± 14.82	69.13 ± 12.78	64.71 ± 12.25	60.74 ± 16.60	63.93 ± 13.66	65.69 ± 11.55	0.306	0.194

FVC: forced vital capacity; %FVC: percentage of predicted forced vital capacity; FEV1: forced expiratory volume in one second; %FEV1: percentage of predicted forced expiratory volume in one second; MVV: maximum voluntary ventilation; %MVV: percentage of predicted maximum voluntary ventilation; *p*. Inter: *p* value of interaction effect from mixed ANOVA; ES: effect size; η2: eta squared.

Data are represented as mean ± SD.

**Fig 2:**
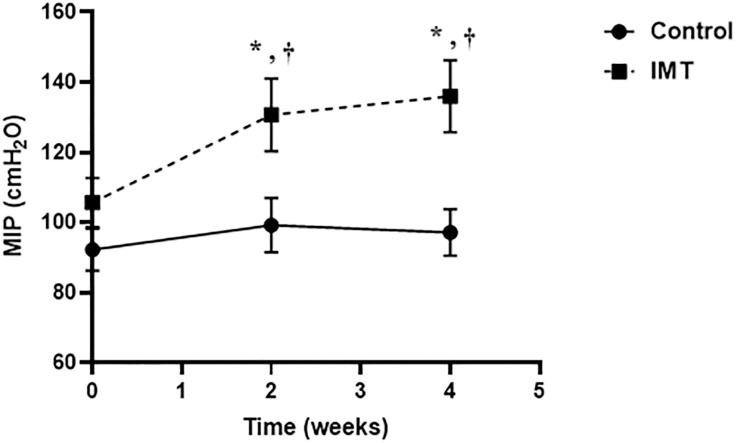
The effect of inspiratory muscle training (IMT) on maximal inspiratory pressure (MIP, cmH₂O) following the 2- and 4-week intervention periods (Time, weeks). Data are represented as mean ± SE. * Differences from baseline were analyzed using repeated measures ANOVA with Bonferroni’s post-hoc test. † Differences from the control group were analyzed using independent t-test.

Interestingly, the enhancement of MIP was found in the IMT group at week 2 and week 4 after intervention when compared with control group and increase from baseline (Fig 2). The separate analyses of repeated measures ANOVA and independent t-test are presented in the supporting information.

### Effects of inspiratory muscle training on heart rate variability in obese young adults

The present study found that the time-domain measures, including SDNN, SDANN, ASDNN, and rMSSD, did not differ significantly for any factor (time or group). Interestingly, the IMT group showed a significant reduction in the LF/HF ratio at weeks 2 and 4 after the intervention (p < 0.05), as shown in Table 3. This decrease suggests a potential improvement in sympathovagal balance, although its clinical significance remains uncertain.

**Table 3:** Parameters of heart rate variability in the control group and obese young adults following the 2 and 4-week intervention period.

HRV Parameters	Control group			IMT group			Mixed ANOVA	
	Baseline	Week 2	Week 4	Baseline	Week 2	Week 4	*p.* Inter	ES (partial η2)
*Time domain*								
SDNN (ms)	149.55 ± 27.45	144.44 ± 34.96	141.22 ± 33.68	152.70 ± 27.10	147.00 ± 18.01	132.80 ± 31.52	0.425	0.101
SDANN (ms)	136.33 ± 28.32	130.67 ± 36.06	128.55 ± 29.91	142.00 ± 29.39	138.50 ± 22.61	132.14 ± 25.55	0.597	0.076
ASDNN (ms)	68.11 ± 17.56	66.00 ± 18.87	64.33 ± 21.27	64.30 ± 12.15	61.30 ± 9.64	61.40 ± 12.58	0.928	0.009
rMSSD (ms)	39.77 ± 9.44	37.67 ± 9.46	36.55 ± 11.41	42.63 ± 11.66	39.90 ± 9.90	41.30 ± 13.57	0.846	0.021
*Frequency domain*								
LF (ms^2^)	27.58 ± 9.22	27.58 ± 10.29	26.63 ± 10.04	25.60 ± 5.88	24.24 ± 4.82	24.32 ± 5.28	0.865	0.018
HF (ms^2^)	18.07 ± 5.80	17.49 ± 6.01	16.59 ± 7.35	18.95 ± 4.83	17.96 ± 4.88	18.28 ± 5.69	0.789	0.029
LF/HF ratio	1.53 ± 0.19	1.58 ± 0.19	1.69 ± 0.35	1.47 ± 0.20	1.32 ± 0.12^*^	1.28 ± 0.17^*^	0.017	0.421

SDNN, standard deviation of all normal sinus R-R intervals in the entire 24-h recording; SDANN, standard deviation of all averaged normal sinus R-R intervals for all 5-min segment in the 24-h recordings; ASDNN, average of the standard deviations of all R-R intervals for all 5-min segments in the 24-h recordings; rMSSD, root mean square of the mean of the squared differences of two consecutive R-R intervals; LF, low frequency power; HF, high frequency power; IMT, inspiratory muscle training. *p*. Inter: *p* value of interaction effect from mixed ANOVA; ES: effect size; η2: eta squared.

Data are represented as mean ± SD.

*p < 0.05 difference between groups.

## Discussion

In this study, we demonstrated that 4 weeks of IMT effectively improved both the HRV and inspiratory muscle strength, as indicated by a decrease in the LF/HF ratio and an increase in MIP in obese young adults. However, IMT did not mitigate the negative effects of obesity on lung function parameters (FVC, FEV1, and FEV1/FVC) and inspiratory muscle endurance (MVV).

A previous study has shown that 4–12 weeks of intervention may be an appropriate duration for respiratory muscle training [32]. Although 4 weeks of IMT improved FVC, FEV1 and MVV in athletes and non-athletes [33,34], these beneficial effects were not observed in overweight and obese adults [20]. Consistent with those findings, 4-weeks of IMT in our study did not attenuate the negative effect of obesity on the pulmonary function parameters, which could be due to the changes in structural and mechanical properties caused by excessive fat accumulation within the thoracic, abdominal cavities and upper airways. The excessive fat accumulation in those compartments could alter the mechanical properties of the lungs and chest wall by reducing their compliance, which could be reversed by weight loss [35]. In addition, a previous study reported that 12 weeks of IMT in morbidly obese individuals enhanced only forced inspiratory volume in one second (FIV1) and reduced extra-thoracic obstruction. [17]. Although longer IMT durations (8–12 weeks) have demonstrated more substantial improvements in pulmonary function, recent studies have shown that even a 4-week protocol can significantly enhance inspiratory muscle strength and sympathovagal balance in overweight and elderly populations [20,22,36]. Therefore, the short training period in the present study might not be enough time to see any significant effects on pulmonary function in these obese participants. Future studies should compare different IMT durations and intensities to identify optimal training regimens. Additionally, the effect of a combination of a short period of IMT intervention with weight loss on dynamic lung function requires further elucidation. As reported previously, respiratory muscle endurance training (REMT) with a load of 60–80% of MVV for 3–4 times per week could improve MVV in obese patients [37]. However, the current study used a different method, guided by a previous study [20] by setting 55% of MIP as the training intensity for 4 weeks. This may explain why we did not observe the significant improvements in this parameter. Thus, these findings suggested that obese subjects need more extended periods of time and training intensity to improve pulmonary function and respiratory muscle endurance. On the contrary, the effects of the training were observed in the IMT group at week 2 and week 4 which is consistent with previous studies [20,24].

To our knowledge, this study is the first to examine the effects of a 4-week IMT program in obese young adults on HRV. Previous studies found that obese populations had changes in cardiac autonomic modulation, as indicated by increased sympathetic activity and decreased vagal tone [38,39]. In our study, the time domain including SDNN, SDANN, ASDNN and rMSSD did not change after the IMT program in either group, which is consistent with previous reports [30,40]. Although the LF/HF ratio significantly decreased following IMT, time-domain markers such as rMSSD and SDNN did not show significant changes. Therefore, the observed improvement may reflect enhanced sympathovagal balance rather than a definitive increase in vagal tone. While prior research has demonstrated that IMT can improve cardiac autonomic modulation, these findings have primarily been observed in clinical populations with chronic conditions [22,19,41]. Evidence supporting such effects in obese young adults remains limited. Therefore, although the significant reduction in the LF/HF ratio in this study suggests a possible improvement in sympathovagal balance, the lack of changes in time-domain HRV parameters and variability in prior findings [30,40] warrant cautious interpretation. Moreover, while the observed improvements in inspiratory muscle strength and LF/HF ratio were statistically significant with moderate effect sizes, the clinical significance of these changes remains to be confirmed. Further studies are needed to determine whether such changes translate into measurable functional or cardiovascular benefits in obese young adults. The lack of changes in time-domain HRV measures (SDNN, SDANN, ASDNN, rMSSD) after the 4-week IMT program in obese young adults could be attributed to several factors. First, the short duration of the intervention may not have been sufficient to induce significant autonomic adaptations. Second, the pre-existing autonomic imbalance in obese individuals, characterized by increased sympathetic activity and reduced vagal tone, may require longer or more intensive interventions to show improvements. Additionally, IMT primarily targets respiratory muscles, and its effects on cardiac autonomic modulation may be more gradual or indirect. Individual variability in response to IMT and the potential insensitivity of time-domain measures to subtle autonomic changes may have further contributed to the lack of observed improvements in HRV.

A recent systematic review demonstrated that an IMT program at an intensity of 30% MIP, for 7 sessions per week for 8 weeks resulted in decreased cardiac sympathetic modulation (LF) and increased parasympathetic modulation (HF) in patients with hypertension, heart failure, and diabetes mellitus [41]. Rodrigues and colleagues demonstrated that 50% of MIP for 4 weeks promoted inspiratory muscle strength, increased HF and decreased LF/HF ratio in elderly women [22]. In the present study, with only 4 weeks of IMT with a load 55% of MIP, we did observe the significant improvements of HRV as indicated by a reduction in LF/HF ratio at week 2 and week 4. Although a longer IMT duration may yield more robust outcomes, our findings demonstrate that a 4-week protocol is sufficient to elicit moderate-to-large improvements in inspiratory muscle strength and sympathovagal balance. This is consistent with previous studies that showed benefits of short-term IMT [20,22,42,43]. However, future studies should compare different durations to determine the optimal training length. The positive effects of IMT on HRV could be explained by IMT inducing the alterations in respiratory patterns, which are a key modulator of ANS as well as other reflex control systems, includes baroreceptors and chemoreceptors [44,45] and consequently enhance cardiac vagal modulation [30,46,47]. Baroreflex sensitivity, which influences HRV through central autonomic integration, may have contributed to the reduction in LF/HF ratio. Although not directly assessed in this study, this mechanism warrants consideration in future research. However, the results of a recent study reveal that IMT did not change baroreflex sensitivity in healthy elderly women [36]. Thus, the beneficial effects of IMT on HRV, the physiological mechanisms involved in the autonomic adaptations require further investigation.

Due to the non-randomized design, there is a potential for selection bias and unbalanced distribution of unmeasured confounding factors, despite matching participants by age and sex. Moreover, although participants were matched for these variables, other unmeasured confounders such as physical activity level, metabolic status, or systemic inflammation may have been unevenly distributed and could have influenced the outcomes. Several limitations should be considered. First, although the sample size was calculated based on power analysis, the relatively small number of participants may limit the generalizability of the findings. Future studies with larger and more diverse populations are required to validate and extend these results. Second, the small sample size, subgroup analyses were not feasible. As individual variability in physiological responses to IMT may exist, future studies with larger cohorts are needed to explore differential effects based on sex, baseline fitness, or autonomic status. Third, the control group did not undergo sham or low resistance breathing training, raising the possibility of placebo effects in the IMT group. Including a sham training group in future studies would help control expectancy bias. Fourth, although participants were instructed to maintain their usual lifestyle habits, potential confounding factors such as physical activity levels and dietary intake were not controlled. This limitation should be considered when interpreting the observed changes in autonomic function. In particular, the lack of monitoring of participant’s physical activity levels and body weight changes during the intervention period may have introduced unmeasured confounding especially concerning HRV and respiratory outcomes. Additionally, physical activity, which was not formally assessed in this study, may contribute to the variability in HRV and inspiratory muscle strength outcomes. Regular exercise is known to promote vagal modulation, improve sympathovagal balance, and enhance respiratory muscle performance through neuromuscular adaptation [48,49]. Therefore, uncontrolled variation in participants’ physical activity levels may have influenced the results and should be addressed in future studies. Lastly, while the reduction in LF/HF ratio suggests enhanced vagal modulation, other physiological mechanisms such as altered respiratory patterns, baroreflex sensitivity, and chemoreceptor-mediated reflexes may also contribute to the observed changes in HRV. These mechanisms warrant further investigation to fully understand the autonomic effects of IMT.

## Conclusion

In summary, a 4-week inspiratory muscle training program modestly improved inspiratory muscle strength and reduced the LF/HF ratio in obese young adults. However, further studies are warranted to determine whether these changes translate into clinically meaningful benefits.

## Supporting information

S1 Table Continuous data corresponding to Fig 2. The effect of inspiratory muscle training (IMT) on maximal inspiratory pressure (MIP, cmH₂O) after 2- and 4-week intervention periods. (PDF)

S2 Table Repeated measures ANOVA summary for within-subject effects of time on maximal inspiratory pressure (MIP) in each group. The IMT group showed a significant effect of time on MIP (p = 0.004), while the control group did not (p = 0.103). Partial eta squared (η²) indicates a large effect size in the IMT group.
(PDF)

S3 Table Pairwise comparisons of maximal inspiratory pressure (MIP) across three time points (week 0, week 2, and week 4) within the control and IMT groups. Significant increases were observed in the IMT group from week 0 to week 2 (p = 0.03) and week 0 to week 4 (p = 0.008). No significant changes were found in the control group.
(PDF)

S4 Table Independent t-tests comparing maximal inspiratory pressure (MIP) between IMT and Control groups at each time point. At baseline (Week 0), there was no significant difference in MIP between groups (p = 0.15). However, significant between-group differences were observed at week 2 (p = 0.02) and week 4 (p = 0.004), with the IMT group showing greater MIP gains.
(PDF)
