# Supplementary material for: Inspiratory muscle training improves heart rate variability and respiratory muscle strength in obese young adults
Source: PLoS One. 2025 Aug 20;20(8):e0329623. doi: 10.1371/journal.pone.0329623 (PMC12367178; doi:10.1371/journal.pone.0329623)
Supplement: S1 Table — (PDF) [file pone.0329623.s001.pdf]

S1 Table. Continuous data corresponding to Fig 2. The effect of inspiratory muscle training (IMT) on maximal inspiratory pressure (MIP, cmH_2O) after 2- and 4-week intervention periods.

Subjects	Groups	Week0	Week2	Week4
1	Control	94	101	108
2	Control	73	75	82
3	Control	124	144	134
4	Control	105	104	106
5	Control	103	127	117
6	Control	70	77	82
7	Control	96	94	90
8	Control	72	86	77
9	Control	93	85	78
10	IMT	100	183	186
11	IMT	75	110	103
12	IMT	118	135	129
13	IMT	107	136	137
14	IMT	83	95	114
15	IMT	100	94	107
16	IMT	140	173	187
17	IMT	89	126	130
18	IMT	123	124	131
19	IMT	116	124	136

Groups	Control			IMT		
	Week 0	Week 2	Week 4	Week 0	Week 2	Week 4
**Mean**	92.22	99.22	97.11	105.10	130.00	136.00
**SE**	5.98	7.71	6.69	6.26	9.26	9.20
