# Supplementary material for: Inspiratory muscle training improves heart rate variability and respiratory muscle strength in obese young adults
Source: PLoS One. 2025 Aug 20;20(8):e0329623. doi: 10.1371/journal.pone.0329623 (PMC12367178; doi:10.1371/journal.pone.0329623)
Supplement: S2 Table — The IMT group showed a significant effect of time on MIP (p = 0.004), while the control group did not (p = 0.103). Partial eta squared (η²) indicates a large effect size in the IMT group. (PDF) [file pone.0329623.s002.pdf]

S2 Table. Repeated measures ANOVA summary for within-subject effects of time on maximal inspiratory pressure (MIP) in each group. The IMT group showed a significant effect of time on MIP ($p = 0.004$), while the control group did not ($p = 0.103$). Partial eta squared (η^2) indicates a large effect size in the IMT group.

**Groups**	**Effect**	**p-value**	**partial η2**
Control	Time	0.103	0.247
IMT	Time	**0.004***	0.585
