# Supplementary material for: Inspiratory muscle training improves heart rate variability and respiratory muscle strength in obese young adults
Source: PLoS One. 2025 Aug 20;20(8):e0329623. doi: 10.1371/journal.pone.0329623 (PMC12367178; doi:10.1371/journal.pone.0329623)
Supplement: S3 Table — Significant increases were observed in the IMT group from week 0 to week 2 (p = 0.03) and week 0 to week 4 (p = 0.008). No significant changes were found in the control group. (PDF) [file pone.0329623.s003.pdf]

S3 Table. Pairwise comparisons of maximal inspiratory pressure (MIP) across three time points (week 0, week 2, and week 4) within the control and IMT groups. Significant increases were observed in the IMT group from week 0 to week 2 (p = 0.03) and week 0 to week 4 (p = 0.008). No significant changes were found in the control group.

Groups	Time comparison	p-value
Control	Wk0 vs. Wk2	0.25
Control	Wk0 vs. Wk4	0.53
Control	Wk2 vs. Wk4	1.00
IMT	Wk0 vs. Wk2	**0.03***
IMT	Wk0 vs. Wk4	**0.008***
IMT	Wk2 vs. Wk4	0.166
