# Supplementary material for: Inspiratory muscle training improves heart rate variability and respiratory muscle strength in obese young adults
Source: PLoS One. 2025 Aug 20;20(8):e0329623. doi: 10.1371/journal.pone.0329623 (PMC12367178; doi:10.1371/journal.pone.0329623)
Supplement: S4 Table — At baseline (Week 0), there was no significant difference in MIP between groups (p = 0.15). However, significant between-group differences were observed at week 2 (p = 0.02) and week 4 (p = 0.004), with the IMT group showing greater MIP gains. (PDF) [file pone.0329623.s004.pdf]

S4 Table. Independent t-tests comparing maximal inspiratory pressure (MIP) between IMT and Control groups at each time point. At baseline (Week 0), there was no significant difference in MIP between groups ($p = 0.15$). However, significant between-group differences were observed at week 2 ($p = 0.02$) and week 4 ($p = 0.004$), with the IMT group showing greater MIP gains.

Time points	Mean difference (IMT-Control)	p-value
Week 0	12.88	0.15
Week 2	30.78	**0.02***
Week 4	38.89	**0.004***
